# Theoretical foundations of Progressive Motor Training (PMT) for Phantom Limb Pain

**DOI:** 10.1186/s12984-025-01790-x

**Published:** 2026-01-06

**Authors:** Max Ortiz-Catalan

**Affiliations:** 1 Prometi Pain Rehabilitation Center, Vinnytsia, Ukraine; 2 Center for Complex Endoprosthetics, Osseointegration, and Bionics, Kyiv, Ukraine

## Abstract

Phantom Limb Pain (PLP) remains highly prevalent despite decades of clinical and experimental interventions. While pharmacological and surgical approaches address acute or nociceptive components, chronic neuropathic PLP persists, and theoretical models often lack testable predictions. Here, I present the theoretical basis for Progressive Motor Training (PMT), a novel treatment derived from the Stochastic Entanglement hypothesis. This hypothesis proposes that PLP arises from maladaptive recruitment of underutilized somatosensory and motor circuits after amputation, rather than cortical reorganization or visual feedback deficits. PMT combines motor imagery and motor execution to maximize recruitment of the affected motor circuitry in a progressive, comprehensive, and adaptive manner. It is designed to overcome limitations of existing motor training therapies, such as Mirror Therapy and Phantom Motor Execution, by being applicable across amputation levels and clinical contexts, including early post-surgical stages where current technology-based interventions are impractical. Therefore, PMT can help to preserve phantom movement and hypothetically reduce PLP incidence when applied early. Overall, PMT offers a theoretically grounded, resource-efficient, and clinically flexible alternative for both treatment and prevention of PLP. Its efficacy alleviating PLP now requires systematic empirical evaluation.

## Background

Phantom Limb Pain (PLP) is a common condition after limb amputation, and despite a myriad of treatments available [1], its prevalence has remained over 60% for the last 50 years [2, 3]. Pharmacological approaches have proven useful in acute, but not in chronic PLP, and can carry considerable side effects [4]. Recent surgical developments in which the severed nerves are provided with biological targets have shown promising results alleviating neuroma pain and nociceptive induced PLP [5]. However, they have been less successful with chronic neuropathic PLP. The underlying mechanisms producing and maintaining PLP, beyond nociception from the severed nerves, remain unclear [6]. This might be the reason for which therapeutic approaches are frequently derived from intuition and often lack theoretical justification.

Mirror Therapy [7], one of the most common therapies for PLP, exemplifies the aforementioned situation and illustrates the challenges in treating PLP. Mirror Therapy has been shown to achieve near-complete alleviation in randomized controlled trials (RCTs) [8], however, widescale implementation with similar outcomes has not been as successful [9]. This is arguably because of the lack of a standardized protocol [10], which one can argue reflects the lack of a clearly understood theoretical basis. Here, I present the theoretical basis of a novel treatment for PLP, namely Progressive Motor Training (PMT), with the intention to aid on its scientific exploration and clinical implementation.

### Hypothesis on the pathogenesis of PLP

Different ideas have been proposed for the pathogenesis of PLP. However, these are rarely postulated as scientific hypotheses lacking explicit testable predictions to disprove them. Such ideas include time to deafferentation, pain memory (pre- to post-amputation pain relationship), atmospheric mediation (PLP reportedly higher in cloudy days), and maladaptive plasticity [11]. The term “maladaptive plasticity theory” is commonly found in the PLP literature despite that it has never been articulated as a scientific theory, or as a hypothesis to begin with. The term is often associated with changes in cortical representation first described in a seminal article by Flor et al. [12], in 1995, and with a correlation, or lack thereof, with PLP [13]. The relationship between cortical changes and PLP has kept the status of correlation without explicit mechanisms for causality. This idea is lately referred as a “model” and continues to be used to provide a sense of explanation, although concrete testable predictions have yet to be postulated.

Scientific hypotheses on PLP explicitly formulated as such include Harris’ sensorimotor incongruence hypothesis (1999) [14]. Harris argued that PLP arises from a mismatch between motor intent and expected sensory feedback. For instance, a person attempting to move their missing limb does not see the corresponding action taking place, which results in incongruence and, according to the hypothesis, pain. Harris emphasized the role of vision and cortical changes, justifying the use of a mirror in Mirror Therapy by stressing the importance of anthropomorphic visual feedback. An implicit prediction of this hypothesis is that attempting to move the phantom limb should provoke PLP, owing to the absence of corresponding sensory feedback. However, subsequent studies have consistently found the opposite: patients who are able to generate phantom movements typically report less PLP [15–18].

More recently, Weiss et al. (2022) reformulated Harris’ line of reasoning within a predictive coding framework [19], proposing that PLP results from persistent prediction errors when motor commands or stimulation of neighboring body regions activate the cortical representation of the missing limb in ways that diverge from expected outcomes. These unresolved errors, they suggest, are processed by the salience network, which assigns them aversive salience and thereby contributes to the experience of pain. Their hypothesis faces the same empirical challenge as Harris’: if unresolved prediction errors from phantom motor commands were the primary driver of PLP, then individuals capable of repeated phantom movements should experience more pain, whereas in practice the opposite trend is observed [15–18]. Their framework has so far been explored mainly through visual paradigms such as mirror illusions and virtual reality manipulations, leaving the role of somatosensory circuits underdeveloped.

In 2018, I introduced the Stochastic Entanglement hypothesis limiting the mechanism involved with pathologic pain processing primarily to somatosensory and motor circuits [11]. I argued that vision, in particular anthropomorphic visual feedback, is not a major driving factor of PLP, and pain alleviation employing Mirror Therapy can be attributed to motor intent and not to “tricking the brain” into believing the missing limb is present through a visual illusion. Moreover, I highlighted the unresolved explanatory gap in the idea that changes in cortical representation influence PLP as often assumed in the Maladaptive Plasticity model. This is the open question of how changes in a part of the brain that do not seem to trigger pain, result in PLP. The fact that cortical stimulation rarely results in PLP [20], and the absence of neuropathic pain in people whose cortical representations have been altered through purposeful and extensive motor training, such as in soccer players or musicians [21, 22], raise doubts about a direct causal relationship between changes in cortical representation and pain. In contrast, the Stochastic Entanglement hypothesis is not limited to the cortex and considers somatosensory and motor circuits throughout the neural axis, accounting for the effect of spurious activation of these underutilized neural resources after an amputation.

A prediction derived from the Stochastic Entanglement hypothesis is that the incidence of PLP must be statistically higher in upper limb than in lower limb amputations, because more neural resources are involved in the former. It also predicts that PLP treatments employing virtual reality (VR) in which the phantom limb is not engaged in active movements, will be less effective than those where the phantom limb is actively involved. For instance, controlling the interactions in a VR environment through the contralateral unimpaired limb, will be less effective than engaging the affected limb as the source of control. The Stochastic Entanglement hypothesis emphasizes somatosensory as opposed to visual feedback, and if correct, would render expensive equipment such as head-mounted displays unnecessary for long-term PLP relief. Moreover, it predicts that blind individuals who suffer an amputation will present similar incidence of PLP, and can be treated using motor and somatosensory training, despite the lack of vision. Consequentially for clinical practice, the Stochastic Entanglement hypothesis predicts that purposeful and extensive engagement of the somatosensory and motor neural resources of the affected limb will result in pain alleviation by preventing them from contributing to its pathological processing, for example by strengthening the network salience against spurious firing of underoccupied neurons that might trigger pain. This is the hypothesized working mechanism of Phantom Motor Execution (PME) [11], a therapy in which phantom motor intention is predicted using myoelectric signals from the residual limb and displayed in real-time in virtual or augmented reality environments (VR/AR) [23].

### Challenges with modern motor training therapies

In PME, the possibility to extract phantom motor volition providing real-time feedback in VR/AR, allows for the use of serious gaming, which in turn increases the intensity of phantom motor training [24]. Theoretically, it is in the increased volume of mindful phantom motor training where the therapeutic effect of PME resides, and therefore MPR and VR/AR are simply technological tools to achieve said goal, in the same way that a mirror facilitates motor training in Mirror Therapy, albeit at a lesser volume of training than when employing serious gaming with digital technologies.

PME has been found effective in chronic intractable PLP [24]. By promoting phantom motor training, PME aims to enable the patient to (re)gain control over the phantom limb. Neurophysiologically, it implies activating the relevant central and peripheral circuitry involved in motor control of the missing limb, from motor planning to producing action potentials travelling down to muscles in the residual limb, as well as through the nerves severed by the amputation which would have reached the missing muscles if present. In principle, PME can be achieved without any technological assistance, simply by attempting to execute phantom movements. However, in practice, exercising phantom movements in this manner is tedious and easily abandoned, arguably because there is no timely feedback for the patient to gauge the resulting intended movements, and because the phantom limb is often perceived as locked in position or “frozen”. PME is conducted to a certain degree in Mirror Therapy, and the extent at which PME is performed depends on how much patients involve their phantom limb in the intervention. As mentioned before, the Stochastic Entanglement hypothesis predicts that PLP relief in Mirror Therapy depends on the degree patients attempt to execute phantom movements and not on the anthropomorphic visual feedback produced by the mirror.

In addition to lacking a standardized treatment protocol [10], an issue with Mirror Therapy is that patients can ignore their phantom limb throughout a whole therapy session, without the therapist even realizing it because there is no observable feedback regarding the involvement of the phantom limb. In PME, myoelectric pattern recognition (MPR) allows for real-time feedback to be observed through mixed reality, but it requires for electrodes to be placed in the residual limb, electromyography acquisition hardware, and processing equipment to compute the decoding algorithms. Critically, it requires muscles in the residual limb to be under volitional control of the patient and that their activity can be captured, which is its major feature and drawback. The lack of a residual limb (e.g., shoulder disarticulation), the presence of nerve injuries (e.g., brachial-plexus injuries), or excessive or compromised soft tissue (e.g., open wounds), renders electromyography unfeasible. PMT was devised to overcome these issues, and to meet the unsatisfied need for earlier treatment (for instance, soon after surgery).

### The origins of Progressive Motor Training

In a recent clinical trial, it was found that PME reduced PLP by over 60%, similarly to Phantom Motor Imagery (PMI) guided by VR/AR but without MPR [25]. After further examination, it was discovered that patients in the PMI group trained with more movements, and of higher complexity, than the PME group. This was because participants in the PMI group could practice movements involving several of the missing joints right from the first session, whereas participants in the PME group were constrained by the MPR learning process, and spent most sessions training with simpler movements involving only a couple of the missing joints. I hypothesize that whereas the PMI group engaged larger portions of the cortex imagining complex movements, the PME group recruited subcortical areas executing simpler ones, and the reason for which both therapies achieved similar pain reduction was because the net engagement of the affected motor circuitry was similar between the two. If true, it would correspond with the predicted outcome under the Stochastic Entanglement hypothesis. **Testable prediction #1**: Activation volume of neural resources engaged in PME and PMI will be similar in a brain imagining study comparing these treatments using variable complexity of movements (complexity of movements constrained in PME to the participants’ proficiency in MPR and unconstrained in PMI, as done in the aforementioned RCT [25]). **Testable prediction #2**: In a RCT comparing PME and PMI using the same movements complexity and training volume, PME will show higher pain relief.

Consequently, I devised PMT to take advantage of both PMI and PME treatments. In PMT, motor imagery is used to recruit a larger portion of the cortex by involving complex movements which cannot be yet executed by the patient, while also employing PME to recruit subcortical areas when training those that can be executed. Patients commonly report not being able to move their phantom limb except for a couple of movements with limited speed or range of motion [26]. Rather than having patients only imagining movements that can already be executed, and thus not engaging subcortical areas as in PMI, patients in PMT execute movements they can and only imagine those they cannot execute. **Testable prediction #3**: PMT will result in higher PLP reduction than PMI in a randomized controlled trial.

A fundamental assumption in PMT is that by using imagination to enable execution, the patient can use the practice gained through imagination to “unlock” the execution of more complex movements over time. **Testable prediction #4**: In a controlled trial between PMT and PME without EMG decoding (MPR), participants in the PMT group will achieve higher control over the phantom limb and consequentially report less PLP.

A major driver for the creation of PMT was my humanitarian work conducted in Ukraine, where the Russian full-scale invasion has resulted in over 100,000 people with amputations. War injuries are often complex and go beyond isolated amputations. These patients are often forced to spend several months in hospital beds with limited rehabilitation options and pain management limited to opioids, which are often ineffective over time. PMT was developed to be able to treat these patients closer to the time of injury and before they are released to a rehabilitation center, which can take several months. PME in these patients is unfeasible given the presence of unhealed wounds and other injuries. Even the placement of a mirror can often be impractical given the limited mobility in these patients. Moreover, the number of nurses and rehabilitation specialists is often insufficient to treat the high number of casualties of war. PMT was therefore purposely designed to be efficient regarding time and resources, and to be applicable to all stages after amputation, in all amputation levels and conditions of the residual limb.

### Progressive Motor Training (PMT)

Fundamentally, PMT consists of using motor imagery to achieve motor execution, allowing for comprehensive and progressive recruitment of the affected motor neural resources. The patient is asked to attempt to execute a movement, and if unsuccessful, the patient is asked to imagine the movement instead. A PMT training session is guided by VR or AR environments that display a virtual limb performing movements of the amputated joints, which the patient must follow by imagination or execution. PMT main features include phantom limb training that is guided, structured, and progressive in complexity, and thus difficulty. In PMT, motor imagery is requested when execution is not possible, while keeping track of the ratio of imagined and executed movements to determine the patient’s phantom motor skills. The phantom motor skill level is then used to modulate the ratio of simple and complex movements the patient must train with. The proportion of complex movements is adaptive and dependent on the patient’s evolving phantom motor skills.

## Discussions

Interesting developments often come out from serendipitous findings. I did not expect PMI to relieve PLP in the same degree as PME because previous studies where patients were asked to simply imagine phantom movement reported negative results [8]. In fact, motor imagery alone had been discouraged as a treatment of PLP [27]. However, outcomes often hinged on implementation details. I designed the particular implementation of PMI to be as fair as possible of a comparison with PME considering the digital technologies employed, which inadvertently resulted in a more intense regime of training with respect to the volume of trained movements (number × complexity of movements). This made the volume of trained movements arguably higher than in previous studies employing motor imagery with negative results. Indeed, intensive mental training has been found to reduce PLP and reverse neuropathic changes [28]. PMT structured and progressive design aims to increase training volume over conventional motor imagery, and therefore it is theoretically more likely to reduce pain. As per the Stochastic Entanglement for the pathogenesis of pain, I hypothesize that net neural activity analysis in the PME and PMI trial would have shown that patients in the PMI group engaged larger portions of the cortex because of training with more complex movements, and the PME group would show more activation in subcortical areas that compensate for the less extensive involvement of the cortex given the execution of simpler movements. Unfortunately, no brain imaging studies were conducted in the RCT of PME and PMI [25], so this remains an open question (Testable Prediction #1). On the upside, this new hypothesis along with the need for an earlier treatment for PLP gave birth to PMT.

Anecdotally, most patients report a frozen phantom limb that is considerably difficult to move [26]. However, my group has found that 79% of people who attempt to move their phantom hand can successfully do so within approximately 2.5 h of a brachial plexus blockade, and this number reduces to 57% when movement is attempted within approximately 6 h [29]. Incidentally, only 1 out of 14 participants reported PLP in this study, which supports the idea that preservation of phantom movement can reduce the incidence of PLP, and thus early training to maintain phantom movement, which is easier to achieve than later, can prevent PLP appearing in the first place. This is explicitly predicted by the Stochastic Entanglement hypothesis and PMT could be a vehicle to enable it, even during the healing process after an amputation. PMT is theoretically applicable for the treatment and prevention of PLP, and its efficacy is now a matter of empirical experimentation.

The Stochastic Entanglement hypothesis predicts a reduction in the likelihood of PLP in patients who undergo surgical reconstruction at the time of primary amputation, provided that the limb’s sensorimotor circuitry remains functionally engaged and not chronically deprived. A theoretically ideal and clinically feasible preventive strategy would include careful handling of transected nerves during amputation, provision of physiological reinnervation targets to prevent neuroma formation and neural underactivity, and early motor training to maintain phantom limb mobility. Such an approach could be implemented through various surgical procedures, including Targeted Muscle [30–32] and/or Sensory Reinnervation [33–35], or Regenerative Peripheral Nerve Interfaces [36, 37], followed by early motor training interventions such as PMT or Mirror Therapy. Further research is needed to empirically validate which combination of treatments produce the most optimal results.

## Conclusions

Progressive Motor Training (PMT) was developed to provide early Phantom Limb Pain (PLP) treatment and overcome the limitation of Phantom Motor Execution (PME) and Mirror Therapy. It was based on observations gathered through empirical testing of the Stochastic Entanglement hypothesis for the pathogenesis of PLP, and PME clinical investigations. Further experimental work is needed to disprove or support PMT as an effective treatment for PLP.

## Data Availability

Not applicable.
